# RAM cannula with Cannulaide versus Hudson prongs for delivery of nasal continuous positive airway pressure in preterm infants: an RCT

**DOI:** 10.1038/s41598-021-02988-4

**Published:** 2021-12-07

**Authors:** Shravani Maram, Srinivas Murki, Sidharth Nayyar, Sandeep Kadam, Tejo Pratap Oleti, Rajendra Prasad Anne, Saikiran Deshobhotla, Deepak Sharma, Subhash Arun, Praveen Rao Vadije

**Affiliations:** 1 Neonatology, Fernandez Hospital, Hyderabad, Telangana India; 2 Paramitha Children’s Hospital, Kothapet, Hyderabad, Telangana 500 074 India; 3 Neonatology, King Edward Memorial (KEM) Hospital, Pune, Maharashtra India; 4 Pediatrics, All India Institute of Medical Sciences, Hyderabad, Telangana India

**Keywords:** Health care, Medical research

## Abstract

Nasal continuous positive airway pressure (nCPAP) is the standard non-invasive respiratory support for newborns with respiratory distress. Nasal injury is a common problem with the interfaces used. To compare the incidence and severity of nasal injury in neonates with respiratory distress and supported on nCPAP with Hudson prong or RAM cannula with Cannulaide, a semipermeable membrane. This is an open-label, parallel-arm, gestational age-stratified, bi-centric, randomized control trial including neonates between 28 and 34 weeks gestational age and birth weight > 1000 g needing nCPAP. The size of the interface was chosen as per the manufacturer’s recommendation. Of the 229 neonates enrolled, 112 were randomized to RAM cannula with Cannulaide and 117 to Hudson prong. The baseline characteristics were similar. Any nasal injury at CPAP removal was significantly lower in the RAM cannula with Cannulaide group [6 (5.4%) vs. 31 (26.4%); risk ratio—0.77 (95% CI 0.69–0.87); *p* = 0.0001]. The incidence of moderate to severe nasal injury, need for mechanical ventilation within 72 h of age, duration of oxygen, and requirement of nCPAP for > 3 days were similar. For preterm infants on nCPAP, RAM cannula with Cannulaide, compared to Hudson prongs, decreases nasal injury without increasing the need for mechanical ventilation.

Trail registration: CTRI/2019/03/018333, http://www.ctri.nic.in.

## Introduction

Respiratory distress among premature neonates is one of the commonest indications requiring NICU admission. Nasal continuous positive airway pressure (nCPAP), which maintains the functional residual volume of the lung, is used widely to provide respiratory support for neonates. nCPAP use is shown to reduce mortality, need for mechanical ventilation, and respiratory failure^1^.

The nasal interface forms a critical element in nCPAP delivery. Various interfaces used include nasopharyngeal tubes, binasal prongs, and nasal masks. For effective delivery of nCPAP, the interfaces are required to maintain a constant and stable airway pressure. However, the force applied on the delicate tissues of the nares and nasal septum by the interfaces compromises the skin integrity and may result in nasal injury. The reported incidence of nasal injuries with the use of nCPAP ranges from 20 to 100%^2–5^. The spectrum of nasal injuries ranges from blanchable erythema, non-blanching hyperemia, and skin erosion to excoriation, columellar necrosis, and full-thickness skin loss^6^. Lower gestational age (< 30 weeks), lower birth weight (< 1500 g), longer duration of nCPAP, incorrect size, and/or fixation of the interface are the reported risk factors for nasal injury^6^.

Hudson prongs (Hudson-RCI, Temecula, CA) were designed to reduce the nasal trauma associated with the delivery of infant nCPAP. They are soft, anatomically curved prongs available in 6 sizes, allowing greater choices for appropriate sizing. Although they are effective and safe, there is a potential to cause nasal septal pressure injury. The RAM cannula (Neotech, Valencia, CA) was approved by the Food and Drug Administration as a Class I medical device for providing supplemental oxygen with 60–80% nasal occlusion^7^. It was being used off-label to provide nCPAP because of its ease of use and perception of decreased nasal injury^8–11^. The device looks similar to a traditional nasal cannula used to deliver oxygen, but its stiffer design allows a higher flow rate and pressure delivery. As it is made of a soft material, there is lower airflow resistance and a tendency for reduced nasal trauma.

While Hochwald et al. made a similar comparison for preterm neonates receiving NIPPV^12^, there are no adequately powered studies that compared these two interfaces in reducing nasal injuries. As previous physiological and in vivo studies^13–17^ demonstrated concerns on the efficacy of RAM cannula to deliver the set pressures, we used Cannulaide, a hydrocolloid protective barrier which secures cannula in place and provides a seal, in RAM cannula group for snugly fitting the nasal cannula, preventing leak and allowing better delivery of CPAP pressures. We aimed to compare RAM cannula with Cannulaide versus Hudson prong for delivery of nCPAP in preterm neonates born between 28 and 34 weeks gestational age and weighing ≥ 1000 g, in reducing nasal injuries. Extremely low birth weight neonates were not included for concerns of increased failure rates.

## Methods

This randomized controlled trial was conducted from April 2019 to May 2020at two level-3 neonatal intensive care units (NICU) of tertiary care hospitals in India. The study protocol was prospectively registered in the clinical trial registry of India on 29/03/2019 with a registration number of CTRI/2019/03/018333 (available at http://www.ctri.nic.in). The study was approved by ethics committees of Fernandez hospitals, Hyderabad, India and King Edward Memorial Hospitals, Pune, India. The study was not funded by any source. No changes were made to the study protocol or outcomes after registration. Informed consent was obtained from the guardians of all the participants. The study was performed following the declaration of Helsinki on research on human subjects. Neonates with major congenital malformations such as congenital diaphragmatic hernia, tracheo-oesophageal fistula, Pierre-Robin sequence, and choanal atresia were excluded. We also excluded neonates requiring mechanical ventilation at admission to NICU, those with poor respiratory efforts or apnea, worsening shock, suspected or proven persistent pulmonary hypertension of newborn, severe metabolic acidosis (pH < 7.20 and base deficit > 10), severe respiratory acidosis (pH < 7.20 and PaCO_2_ > 60 mm Hg) and massive pulmonary haemorrhage.

Neonates were randomly allocated to nCPAP with either Hudson prongs or RAM cannula with Cannulaide by the primary investigator, using random numbers contained in serially numbered opaque envelopes, after taking informed consent from parent/guardian. Individual randomization was done for each infant in multifetal gestation. Block randomization was done using computer-generated numbers, by a person not involved in the study. Stratified randomization technique was used based on gestational age (28–30 weeks and 31–34 weeks). Recording of basic clinical data of neonates was done before opening the coded envelopes to optimize allocation concealment. During the study period, both the neonatal units used double-lumen bubble CPAP (B-CPAP) for delivering nCPAP to preterm neonates. nCPAP initiation, titration, and weaning were as per the standard protocol of the participating centres.

The primary outcomes of interest were the incidence and severity of nasal injury at the removal of nCPAP. The secondary outcomes included nasal injury score at discharge, need for mechanical ventilation, duration of nCPAP, need for a change of interface, mortality, and other neonatal morbidities.

**Figure 1:**
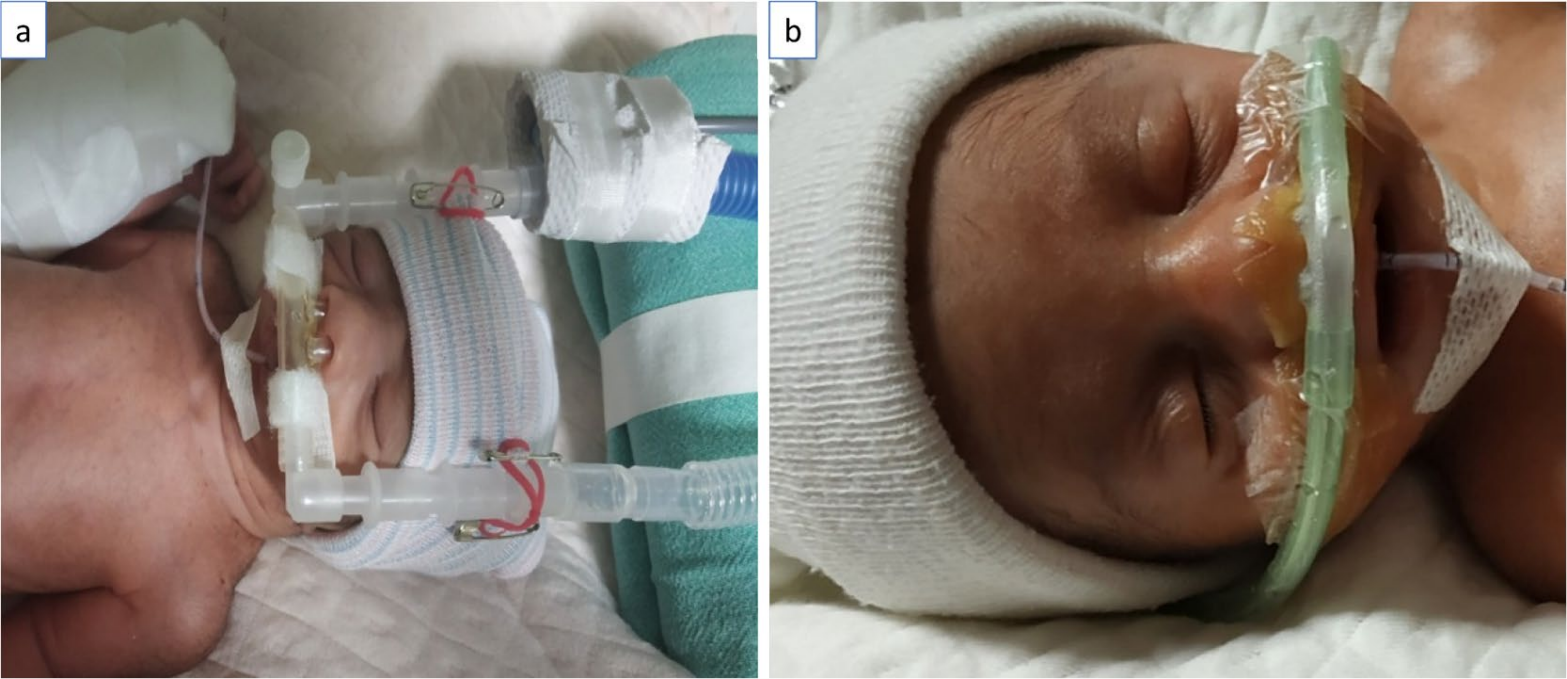
Representative clinical images of Hudson prongs (**a**) and Ram cannula (**b**) as nasal interface for delivering nasal continuous positive airway pressure.

In the Hudson prong group, the neonates were given nCPAP support by using appropriately sized Hudson prongs (as per the manufacturers’ recommendations) till nCPAP was weaned off. The prongs were connected to the nCPAP circuit directly using pins and rubber bands over appropriate-sized bonnets and secured in place with a strip of Velcro tape placed across the infant’s upper lip (Fig. 1). In the RAM cannula group, an appropriately sized RAM cannula as per the manufacturer’s instructions was used for providing nCPAP. A semipermeable membrane (Cannulaide) was used to fix the RAM cannula and to minimize leaks around the nostrils. Cannulaide was prepared by NICU staff using Duoderm (extra thin CGF/Convatec) with two small holes to insert the nasal prongs. As there is no separate expiratory limb to vent out inhaled gases, the infant’s mouth was not actively closed as an important safety measure (Fig. 1).

Routine monitoring of neonate on nCPAP included flow rate, the fraction of inhaled oxygen (FiO_2_), CPAP pressure, bubbling, humidifier temperature, water level, interface size, fixation, the distance between interface and infant nostrils. All enrolled infants were monitored 8th hourly and assessed for nasal injury by the bedside nurse under the supervision of a neonatal fellow who has observed at least 10 nasal injuries correctly. A standard nasal injury score chart^18,19^ was used (Table 1). At the removal of nCPAP, in addition, digital photographs were taken and were later reviewed by a senior neonatologist who was blinded to the study group allocation. Informed consent was taken from parents to publish the clinical photographs.

**Table 1:** Nasal injury assessment score chart.

Tip of nose	0 = normal
	1 = red
	2 = red + indent
	3 = red/indent/skin breakdown
	4 = as above + tissue loss
Nasal septum	0 = normal
	1 = red
	2 = red + indent
	3 = red/indent/skin breakdown
	4 = as above + tissue loss
Nostrils	0 = normal
	1 = enlarged
	2 = enlarged and prong shape
	3 = red, bleeding
	4 = as above + skin breakdown
Nose shape	0 = normal
	1 = pushed up/back but normal
	2 = pushed up and shortened. No normal orientation when prongs removed
Bridge of the nose	0 = normal
	1 = red
	2 = red + indent
	3 = red/indent/skin breakdown
	4 = as above + tissue loss
Upper lip	0 = normal
	1 = red
	2 = red + indent
	3 = red/indent/skin breakdown
	4 = as above + tissue loss

*Scoring*: 0 = no injury, 1–4 = mild injury, 5–6 = moderate injury, > 7 = severe injury.

Weaning from nCPAP was attempted if the infant had good respiratory efforts, minimal or no recessions, no tachypnoea, no apnea/bradycardia, and was on stable or decreasing oxygen requirement for 4 to 6 h. nCPAP was tapered to high flow nasal cannula (HFNC) at nCPAP pressure of 4 cm of H_2_O, FiO_2_ of < 30%, and the neonate was hemodynamically stable, with good respiratory efforts and no apnea/bradycardia. If the infant remained stable on HFNC for at least 6 h, the infant was considered to have completed the study. If the infant did not tolerate HFNC within the 6-h trial period, the infant was restarted on nCPAP using the same nasal interface as before.

Need for mechanical ventilation or non-invasive positive pressure ventilation (NIPPV) (nCPAP failure) was considered if an infant receiving CPAP pressure > 7 cm water had FiO_2_ requirement > 0.6, severe respiratory acidosis, severe metabolic acidosis, increasing work of breathing (Silverman Anderson score/SAS increased by > 2 from baseline) or recurrent/severe apneas (> 4 episodes per hour or need for bag and mask ventilation for any apnea). Change of nasal interface was considered if the infant had a nasal injury score ≥ 4. The nCPAP interfaces were changed in a crossover pattern and then analyzed as per intention to treat analysis.

A previous study noted the incidence of moderate to severe nasal injury of 33% among the Hudson prong group^18^. Assuming the reduction of nasal injury by 50% (an absolute reduction of 16.5%) with RAM cannula with Cannulaide, taking 80% power, 5% alpha error, and 95% confidence interval, we calculated a sample size of 109 neonates in each group in a superiority design (margin of 0.05) to prove our hypothesis.

Descriptive statistics were used to describe baseline variables. Categorical outcome variables were expressed as proportion and analyzed by Chi-square test with continuity correction or Fisher’s exact test, wherever one or more expected cell sizes is less than 5. Estimates of the strength of association were deduced by calculating relative risks with their respective 95% confidence intervals. Continuous variables were expressed as mean (standard deviation) or median (interquartile range) based on their distribution. These variables were first tested for normality utilizing the Kolmogorov–Smirnov test for normality, and analyzed by student ‘t’ test or Wilcoxon rank sign test. All analyses were done using IBM-SPSS v.20 and Microsoft Excel. A *p* value of < 0.05 was considered significant. Binominal regression analysis was performed to identify the predictors of nasal injury.

### Ethical review board

Institutional Ethics Committee, Fernandez Hospital Foundation (Reg. no. ECR/933/Inst/TG/2017); Reference number: 19_2018.

### Informed consent

An informed consent was taken from the infant parents or legal guardian before the enrolment into the study.

## Results

**Figure 2:**
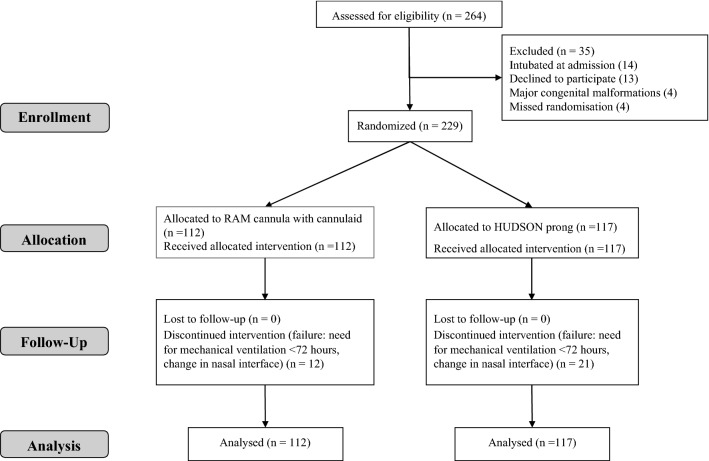
Participant flow chart.

During the study period, 264 preterm infants were assessed for eligibility and 229 infants were enrolled (205 infants from Fernandez Hospital, and 24 infants from King Edward Memorial Hospital). The participant flow is shown in Fig. 2. The baseline characteristics of neonates including gestational age, birth weight, gender, the incidence of intrauterine growth retardation, distribution of infants in the gestational age strata, Apgar scores, and SNAPPE II (Score for Neonatal Acute Physiology-Perinatal Extension, version 2) scores at admission were similar between the two groups. The maternal characteristics of antenatal steroid coverage, mode of delivery, and multifetal pregnancies were also similar. The characteristics of respiratory illness including the severity of respiratory distress (measured by SAS), number of infants with respiratory distress syndrome, surfactant requirement, age at enrolment, FiO_2_ at enrolment, CPAP at enrolment, maximum FiO_2_ required, maximum CPAP required, and age at receiving surfactant were similar between Hudson prong and RAM cannula with Cannulaide groups (Table 2).

**Table 2:** Baseline maternal and neonatal characteristics.

Neonatal characteristics	RAM cannula (n = 112)	Hudson prong (n = 117)	*p* value
Gestationalage in weeks^a^	31.4 ± 1.7	31.4 ± 1.6	0.96
Birth weight in grams^a^	1491 ± 321	1531 ± 394	0.41
IUGR^b^	29 (25.9)	31 (26.5)	0.92
**Gestation age strata**^**b**^			
28–30 weeks	33 (29.5)	34 (29.1)	0.95
31–34 weeks	86 (71)	88 (69)	
Male sex^b^	65 (58)	67 (57.3)	0.91
Apgar score at 5 min^c^	8 (7–8)	8 (8–8)	0.49
SNAPPE-II score^c^	5 (1.25–5)	5 (0–5)	0.87
Any antenatal steroid^b^	110 (98.2)	115 (98.3)	0.97
Caesarean^b^	96 (85.7)	108 (92.3)	0.11
Singleton^b^	57 (50.9)	72 (61.5)	0.25
Respiratory Distress Syndrome^b^	68 (60.7%)	77 (65.8%)	0.42
Age at enrolment (h)^a^	0.48 ± 0.16	0.47 ± 0.09	0.36
SAS score at randomization^c^	5 (5–6)	5 (5–5)	0.35
Post-randomization surfactant^b^	60 (53.6)	67 (57.3)	0.57
FiO_2_ at enrolment^c^	0.3 (0.3–0.3)	0.3 (0.25–0.3)	0.5
Pressure at enrolment^c^	5 (5–5)	5 (5–5)	0.43
Age at 1st dose surfactant (h)^c^	1 (0.75–1)	1 (0.75–1)	0.83
Maximum FiO_2_ on CPAP^c^	0.3 (0.3–0.3)	0.3 (0.25–0.3)	0.54
Maximum CPAP (cmH20)^c^	6 (5–6)	5 (5–6)	0.68

*IUGR* intrauterine growth restriction, *SNAPPE-II* score for neonatal acute physiology with perinatal extension-II, *SAS* Silverman Anderson score, *FiO*_*2*_ fraction of inspired oxygen, *CPAP* continuous positive airway pressure.

^a^Mean ± standard deviation.

^b^n (%).

^c^Median (inter quartile range).

Thirty one infants (26.4%) in Hudson prong group and 6 infants (5.35%) in RAM cannula with Cannulaide group had nasal injury (RR—0.77, 95% CI 0.69–0.87, *p* = 0.0001; Absolute risk reduction—0.21, 95% CI 0.12–0.3; Number need to prevent one injury—4.7, 95% CI 3.32–8.26). In the Hudson Prongs group, 29 infants had a mild nasal injury (score 1–4) while 2 had a moderate nasal injury (score 5–6). All 6 infants in RAM cannula with Cannulaide group had a mild nasal injury. None of the infants suffered from severe nasal injury. Four infants (3.4%) in the Hudson prong group and none of the infants in the RAM cannula group with Cannulaide group had persistence of nasal injury at discharge (RR—0.966; 95% CI 0.93–0.99, *p* = 0.048). Among the 37 infants with nasal injury, 12 (32%), 6 (16%), 9 (24%) and 8 (22%) infants had maximum nasal injury scores of 1, 2, 3, and 4 at the removal of nCPAP, respectively. One infant each had a maximum nasal injury score of 5 and 6. On subjective evaluation, none had skin excoriation, 32 (87%) infants had erythema, 21 (57%) infants had dilation of nostrils and 4 (11%) infants had indentation. All 4 infants with the persistence of nasal injury at the time of discharge had discolouration over the upper lip.

In the Hudson prong group, significantly more neonates required a change of interface as compared to RAM cannula with the Cannulaide group. The duration of nCPAP was significantly longer in RAM cannula with the Cannulaide group. The need for mechanical ventilation within 72 h of age, duration of oxygen therapy, nCPAP requirement for more than 3 days, air leaks, and need for supplemental oxygen at 28 days of life were not different between the two groups (Table 3). The interface was changed for one neonate in the RAM cannula with Cannulaide group because of worsening respiratory distress and for 8 infants in the Hudson prong group for nasal injury (n = 5) or worsening respiratory distress (n = 3).

**Table 3:** Secondary outcomes and neonatal morbidities.

Secondary outcomes	RAM cannula (n = 112)	Hudson prong (n = 117)	RR/mean difference (95% CI)	*p* value
Need for mechanical ventilation in first 72 h of age^a^	11 (9.8)	13 (11.4)	0.97 (0.86–1.09)	0.75
Change of interface^a^	1 (0.82)	8 (6.3)	0.94 (0.89–0.99)	0.02
Duration of CPAP (h)^b^	20 (12–38)	14 (7–24)	9.3 (1.7–17)	0.04
Infants who remained on CPAP by 3 days of age^a^	11 (9.8)	10 (8.5)	0.87 (0.38–1.96)	0.74
Duration of O_2_ (days)^b^	3 (2–6)	3 (2–5)	− 0.13 (− 2.2 to 2)	0.8
CPAP failure (MV < 72 h/change in nasal interface)^a^	12 (10.7)	21 (17.9)	0.91 (0.82–1)	0.12
Culture positive sepsis^a^	8 (7.1)	17 (14.5)	0.92 (0.84–1)	0.07
Patent ductus arteriosus^a^	3 (2.7)	13 (11.1)	0.91 (0.85–0.98)	0.01
Necrotizing enterocolitis^a^	4 (3.6)	6 (5.1)	0.98 (0.93–1)	0.56
IVH grade 3 or more^a^	0	1 (0.9)	0.99 (0.97–1)	0.32
Cystic PVL^a^	0	1 (0.9)	0.99 (0.97–1)	0.32
ROP needing laser^a^	0	3 (2.6)	0.97 (0.94–1)	0.09
Supplemental O_2_ at 28 days^a^	4 (3.6)	5 (4.3)	0.99 (0.97–1)	0.76
Air leaks^a^	3 (2.7)	1 (0.9)	1 (0.98–1.05)	0.3
Mortality^a^	1 (0.9)	4 (3.4)	0.97 (0.93–1.03)	0.19
Transfers to other hospital^a^	3 (2.67)	7 (6)	0.45 (0.12–1.69)	0.24
Discharge from hospital^a^	108 (96.4)	106 (90.5)	1.06 (0.99–1.14)	0.07
Length of hospital stay (days)^c^	17.6 ± 12.8	16.7 ± 11.9	− 0.3 (− 3.3 to 2.7)	0.59
Weight at discharge (g)^c^	1590 ± 220	1587 ± 265	6.2 (− 55 to 66)	0.92
Length at discharge (cms)^c^	42.8 ± 2.5	42 ± 2.9	− 0.24 (− 0.92 to 0.43)	0.35
Head circumference at discharge (cms)^c^	30.1 ± 1.6	30.2 ± 1.7	0.07 (− 0.3 to 0.49)	0.64

*MV* mechanical ventilation, *IQR* interquartile range, *CPAP* continuous positive airway pressure.

^a^n (%).

^b^Median (inter quartile range).

^c^Mean (standard deviation).

The neonatal morbidities (culture-positive sepsis, necrotizing enterocolitis, grade 3 or 4 intraventricular hemorrhage, cystic periventricular leukomalacia, retinopathy of prematurity requiring laser, and mortality) except patent ductus arteriosus were not different between the two groups. The mean length of hospital stay and anthropometry at discharge were comparable between RAM cannula and Hudson prong group (Table 3). Five infants (2.2%) died during the hospital stay and 10 infants (4.3%) got transferred to other hospitals for continuing care. Of these, 4 infants were still on nCPAP at the time of transfer. Two hundred and fourteen infants (93.4%) got discharged from the hospital.

The potential predictors included were gestation at birth, birth weight, antenatal steroid coverage, SNAPPE II score, SA score, duration of nCPAP, nCPAP interface, FiO_2_ at enrolment, and maximum FiO_2_ required during the study. Nasal CPAP interface and duration of nCPAP were significantly associated with nasal injury (Table 4).

**Table 4:** Predictors of nasal injury.

Independent variables	Nasal injury	No nasal injury	aOR (95% CI)	*p* value
Gestation weeks^a^	31.4 ± 1.85	31.4 ± 1.68	0.82 (0.59–1.15)	0.16
Birth weight (g)^a^	1489 ± 388	1516 ± 355	1 (0.999–1.002)	0.26
Antenatal steroid coverage^b^	35 (94.6)	190 (99)	6.73 (0.53–86.5)	0.14
Hudson group as interface^b^	31 (83.7)	86 (44.8)	11.6 (3.89–34.6)	< 0.0001
SNAPPE II score^c^	5 (5–5)	5 (0–5)	0.95 (0.87–1.04)	0.25
SAS at enrolment^c^	5 (5–6)	5 (5–6)	1.25 (0.69–2.25)	0.45
Duration of CPAP (h)^c^	30 (12–76.5)	22 (12–40)	0.97 (0.96–0.98)	< 0.0001
FiO_2_ requirement at the start of CPAP^c^	0.3 (0.3–0.3)	0.3 (0.3–0.3)	1.002 (0.88–1.13)	0.98
Maximum FiO_2_ requirement^c^	0.3 (0.3–0.35)	0.3 (0.3–0.3)	1.005 (0.93–1.08)	0.91

*aOR* adjusted odds ratio, *CI* confidence interval, *SNAPPE II* score for neonatal acute physiology with perinatal extension II, *SAS* Silverman Anderson score, *CPAP* continuous positive airway pressure, *FiO*_*2*_ fraction of inspired oxygen.

^a^Mean (standard deviation).

^b^n (%).

^c^Median (inter quartile range).

## Discussion

In this RCT, we compared the use of RAM cannula with Cannulaide versus Hudson prongs for delivery of nCPAP in preterm infants with birth weight ≥ 1000 g and gestation 28^0/7^–34^6/7^ weeks. Cannulaide was used in fixing RAM cannula for decreasing leaks around the nostrils and delivery of better CPAP pressures. The incidence of nasal injury was significantly lower with the use of RAM cannula with Cannulaide when compared to Hudson’s bi-nasal prongs. Previous studies also reported less nasal injury with RAM cannula. Drescher et al. compared 36 infants on RAM cannula with 36 infants on the nasal mask or bi-nasal prongs in preterm infants born before 29 weeks’ gestation with birth weight < 1500 g^8^. Nasal injury, described as skin and/or mucosal breakdown, was significantly lower in the RAM cannula group (3 vs. 19; *p* < 0.001). They used Duoderm dressing for protecting the skin at pressure points. However, this study was done in a before-and-after study design, with small sample size. In an RCT, Gokce et al. compared RAM cannula (n = 64) with Hudson prongs (n = 62) in preterm neonates (26–34 weeks’ gestation) for non-invasive respiratory support^11^. Incidence of nasal injury (defined as stage I—hyperemia and hemorrhage, stage II—disruption of skin integrity and superficial ulceration, stage III—nasal deformity) was similar between the two groups (8 vs. 7; *p* = 0.83). Contrary to our study methods, Cannulaide was used for short bi-nasal prongs. This study had a small sample size, the nasal injury was the secondary outcome, and the nasal injury assessment concentrated mainly on the septal injury.

In the present study, we included a more robust assessment of the nasal injury. It could be the reason for identifying many mild nasal injuries in our study, especially in the Hudson prong group. The use of Cannulaide, softness of prongs, ease of fixation, and stability may be the reasons for less nasal injury in the RAM cannula group. However, even in the Hudson prong group, very few had moderate/severe nasal injuries. Use of barrier dressing (Duoderm) over the upper lip, training of nurses in the care of infants on nCPAP, nasal injury monitoring, and using objective scoring were the additional methods that would have reduced nasal injury. These findings also emphasize that, under controlled conditions, reduction of moderate to severe nasal injury is possible with any nasal interface.

The other important concern with RAM cannula is the delivery of pressures and nCPAP failures. In the study by Drescher et al., although the neonates on RAM cannula needed higher settings, and had a shorter time to reintubation, there was no significant difference in non-invasive respiratory support failure rates^8^. A significant reduction in the duration of respiratory support and a trend towards reduction of bronchopulmonary dysplasia was noted. In the RCT by Gokce et al., more neonates with RAM cannula needed invasive ventilation in the first 72 h, surfactant administration and also, repeated doses of surfactant^11^. However, there was no increase in the duration of respiratory support and bronchopulmonary dysplasia. Enrolment of smaller infants, a higher threshold for surfactant administration (FiO_2_ > 0.4), use of Cannulaide in the short binasal prong group but not in the RAM cannula group, and the use of lower CPAP pressures (5–6 cm of H_2_O) may be the reasons for higher failure rate in RAM cannula group in that study.

In our study, the lesser need for invasive ventilation/NIPPV in both the groups could be due to early and liberal use of surfactant (FiO_2_ ≥ 30%), use of Cannulaide in RAM cannula group which achieved near 100% nasal seal, enrolment of bigger infants with weight ≥ 1000 g and gestation ≥ 28 weeks and possible use of higher pressures in the RAM cannula group.

Physiological studies showed that RAM cannula had high resistance^15^, pressure delivered was acceptable with leaks < 30%^16^, and it could not deliver set CPAP levels when applied using the manufacturer’s recommended 60–80% nares occlusion, even with closed mouth and full nasal prong insertion^14^. Matlock et al. noted that NIPPV with RAM cannula produced clinically insignificant tidal volumes in preterm neonates between 24 and 34 weeks during non-spontaneous inflations^20^. Sharma et al. compared the pressures delivered by three different nasal interfaces (nasal mask, Hudson prong, RAM cannula) on 30 neonates in each group with gestation 28–34 weeks and birth weight ≥ 1000 g in our centre, during this study period^21^. We found that none of the nasal interfaces delivered oropharyngeal pressures equivalent to set CPAP pressures. Nasal mask delivered pressures best matched to the set CPAP pressures, while RAM cannula delivered the least effective pressures.

The incidence of patent ductus arteriosus requiring medical or surgical intervention was significantly higher in the RAM cannula group. On regression analysis, apart from the interface used, birth weight and need for mechanical ventilation within 72 h were found to be significantly associated with the incidence of PDA. It may emphasize that these infants were sicker, or it could also be a chance finding.

Strengths of this study include (a) use of a standard nasal injury chart that was used previously in published nCPAP studies, (b) meticulous monitoring for nasal injury in a structured format, (c) review of photographs by senior consultant neonatologist who was blinded to the outcome assessment, and (d) use of bubble CPAP as a primary mode of respiratory support, making our results more generalizable and applicable in resource-limited settings. Also, this is the largest study to date comparing RAM cannula with Hudson prong for nasal injury and CPAP failure. Limitations of this study are the exclusion of extremely preterm and extremely low birth weight infants, the risk group with maximum nasal injury and possible detection bias due to reporting of nasal injury using photographs.

## Conclusion

For preterm infants on nCPAP, the use of RAM cannula with Cannulaide compared to Hudson prongs as interface reduces nasal injury with equivalent success rate for delivering nCPAP. However, this requires the use of a semipermeable dressing to provide a complete nasal seal. Duration of nCPAP was found to be associated with nasal injury apart from the interface. Further studies are needed to evaluate RAM cannula in reducing the nasal injuries when compared to a nasal mask, for efficacy in extremely preterm and extremely low birth weight infants.

## Data Availability

The data will be shared by the corresponding author on a reasonable request.
